# Vaccination in the Light of the Royal British Commission

**Published:** 1898-09

**Authors:** M. R. Leverson

**Affiliations:** Fort Hamilton, L. I., N. Y.


### VACCINATION IN THE LIGHT OF THE ROYAL BRITISH COMMISSION.

M. R. Leverson, M. D., Fort Hamilton, L. I., N. Y.

*Meeting of May 16th, 1890.**

Wm. Tebb examined. Qs. 9,452-4. Is President of the London Society for the abolition of Compulsory Vaccination. Has taken interest in the subject during the past twenty years. Q. 9,456. Will give testimony upon the following points:

1. Preliminary statement of the circumstances which first awakened his interest in the subject of vaccination.

2. Results of personal inquiries into the deaths due to re-vaccination of soldiers at Dortrecht, Holland, in 1883.

3. Results of personal investigation into the serious disaster following re-vaccination in the Fourth Regiment of Zouaves in Algiers, in 1880.

4. Other information as to the dangers attending re-vaccination in the army.

5. A vaccine disaster at Rügen, North Germany, as disclosed by the official report of a Government Commission in the Journal of the Imperial Sanitary Commission, Nos. 24 and 26, published at Berlin 1885, and in my correspondence with one of the Commissioners.

6. Particulars received from Dr. James B. Baird, President of the Board of Health, Atlanta, Ga., U. S., concerning a disaster due to re-vaccination in Thomasville, Ga., U. S., in 1882.

7. Parental reasons for non-vaccination as disclosed in the household census returns, the reports of vaccination prose-

---

*As stated in the August number of The Homœopathic Physician, the testimony of Mr. Tebb, because of its exceptional importance, is now published out of the proper order; being made to take precedence of that of Dr. Creighton.

cutions, and the proposal forms of the defense fund of the London Society for the Abolition of Compulsory Vaccination.

8. Details of disaster following vaccination in Villefranche (D'Aveyron), at Motte aux Bois, and in the Department de l'Oise, France.

9. The relation which appears to subsist between vaccination and leprosy in the West Indies and British Guiana, as the result of inquiries made by me in the early part of last year (1889).

*Lastly*. Other evidence which has from time to time come before me as President of the London Society for the Abolition of Compulsory Vaccination.

Q. 9,457. *As to No. 1:* Twenty years ago my wife took my second daughter to our family doctor for vaccination. The operation apparently produced no effect. A few weeks later my wife took the child again; the doctor said: "Madam, I would not recommend you to have the child vaccinated again; vaccination does not prevent small-pox, and it may do the child an injury." This led me to consider the subject. In reading the medical journals my attention was arrested by the contradictions and confusion of medical opinion as to the kind of lymph to be used, the number of punctures necessary, the amount of protection afforded, the necessity and demand for a repetition of the operation, especially during epidemics. Saw article in *Lancet* of July 15th, 1871, stating that early in the epidemic 122,000 *vaccinated* persons had been attacked, of whom 10,000 died. Mr. Tebb described the various prosecutions to which he had been subjected—some refusals by magistrates to listen to the defense in his own cases and those of others; the bigoted remarks of Lord Chief Justice Cockburn on the hearing of his appeai against repeated convictions; the cruel and vindictive persecutions to which he and others had been subjected; a correspondence with Mr. John Bright, who deplored the injustice of the law and his inability to do anything in the matter. He was asked by the Chairman if he was during this time President of the Association for the Abolition of Compulsory Vaccination (Q. 9,459). To which he replied that he was merely an inquirer at the time. In answer to Sir Guyer Hunter he says he was not at the time connected with any of the anti-vaccination societies. After the twelfth summons issued against him the Board of Guardians passed another resolution to continue the prosecutions. He thereupon addressed a letter to the Board, in which he laid before them the extent of the persecution to which he had been subjected, which in pecuniary loss alone amounted to £200 ($1,000), and stated that no amount of continued prosecutions would alter his determination not to submit his child to the risks of injury by vaccination. Also that in Vol. VIII, p. 193, of the *Medical Observer*, 1808, are given 535 cases of small-pox after cow-pox, 150 cases of cow-pox disease. The article in the *Medical Observer* was by Dr. Charles Maclean, London, August 20th, 1810.

*As to No. 2* (Q. 9,465). On August 6th, 1883, he visited Dortrecht, Holland, for the purpose of investigating certain fatalities reported in the Amsterdam *Standard*, but contradicted by other journals. On May 25th, 1883, sixty-eight recruits of the Pontonniers were re-vaccinated with arm-to-arm lymph obtained from Maastricht, South Holland. In a few days seven of the soldiers were found seriously injured in the vaccinated arm, and after acute sufferings three of them succumbed.

Q. 9,467. Details the circumstances and authority on which the above statements were made, showing them to be irrefragable and official. Also that in consequence the Minister of War of Holland (Mr. Weitzel) had issued a circular notifying recruits that vaccination was thenceforth not to be obligatory. Also that these facts were brought to to the notice of Parliament and of the Secretary of War by

Mr. Arthur O'Connor on August 14th, 1883, but up to March 26th, 1890, the British War Department had not altered their practice on the subject.

On June 21st, 1883, Mr. Fabius, in the second chamber of the Holland Parliament, had interpolated the Minister of War in Parliament, and that Mr. Weitzel, after detailing the facts as above set forth, further states that every precaution was taken at the time by the surgeon to secure the safety and efficiency of the operation.

Mr. Tebb also showed that one journal, the *Handeloblad*, and some surgeons had, in the face of the admissions and action of the Minister of War, actually presumed to say "that vaccination had nothing to do with the disaster."

Q. 9,468. Details the abrogation of the regulation enforcing re-vaccination in the Swiss Army on December 26th, 1882. At the International Anti-Vaccination Congress, held at the Hotel de Ville, Berne, a re-vaccinated soldier, Nicholas Gfellen, Mounsingen, was introduced, suffering from caries from re-vaccination. Had been afflicted fifteen years. The case was medically examined by Dr. Boëns, Dr. Oidtmann, Professor Vogt, and other medical men.

Q. 9,472 (Dr. Bristowe). "And this inquiry was held fifteen years after the patient's vaccination?" Answer: "This unfortunate man had suffered fifteen years."

Qs. 9,475-86. Sir Guyer Hunter and Mr. Meadows White inquired into the personal statement (under heading No. 1), and find it very curious that after taking a fee of one guinea for vaccinating his child a medical man should afterwards tell him that vaccination was no protection. By their questionings they bring out more strongly than before the fact that both Mr. and Mrs. Tebb, prior and up to this time, had a strong belief in the efficacy and innocuousness of vaccination.

Qs. 9,486a-91 are attempts to belittle the Dortrecht case. They fail.

Q. 9,492 (to Mr. Bradlaugh). "The Minister of War had given an answer in the Chamber, stating the facts."

Q. 4,993. Mr. Tebb was refused a copy of the official proceedings in relation thereto, Dr. Rutgers stating he was not authorized to send a copy.

Qs. 9,498-9,502. Mr. Meadows White probes the evidence in the Swiss case.

Q. 9,503 (Mr. Picton). "Did not Dr. Oidtmann or Dr. Vogt inquire into the origin of the disease?" "Yes."

Q. 9,504. "And questioned the man?" "Yes; all the medical men seemed deeply interested in comparing the answers of the man with his appearance." Q. 9,474. Both pro-vaccinators as well as anti-vaccinators were present at the Congress.

Q. 9,515 (Mr. Hutchinson). "With reference to the Dortrecht case, do you know whether any report has been made which has found its way into the English medical journals?" "I believe there has been no mention of the case in the English medical journals."*

Down to Q. 9,526 the nature of the evidence for the Dortrecht case is inquired into—it remains unshaken.

Q. 9,527 (By the Chairman). The paper in the eighth volume of the *Medical Observer* is written very strongly, attacking vaccination and vaccinators.

Q. 9,530. *As to No. 3*—The Algiers disaster on December 30th, 1880, fifty-eight young recruits of the Fourth Regiment of Zouaves, stationed at Algiers, were vaccinated and infected with an aggravated form of syphilis.

Ten weeks after the operation the soldiers were sent to hospital. In a month all except six were dismissed, but were soon compelled to return. Syphilis had affected their

*Nor have I been able to find any in the American medical journals. It is one unhappy fact that nearly all the medical journals, by a sort of "conspiracy of silence," refuse to admit into their columns any fact which tells against their fetich—vaccination.—M. R. L.

constitution. Some had ulcers for four or five months; one had not recovered in the eighth month; others had affections of the lips, tongue, or palate. Some showed discoloration of the skin; some had violent headaches; affections of the teeth and gums and of the joints presented themselves to my observation, in addition to the usual symptoms of this dangerous and disgusting malady. Some had decay of the hair of the head, eyebrows, and eyelashes.

Of these sad cases, though the details were published in various English and French journals, not one word appeared in any of the medical journals with exception of a slighting reference to it in the *Lancet* (London) of September 3d, 1881, p. 439.

Q. 9,532. Mr. Tebb described his voyage to Algiers to try and learn the facts and the attempts made both in France and England to suppress them. He quotes Dr. Emile Bertherand, one of the leading physicians of Paris, as saying: "The denials of your chiefs in London of this sinister affair on theoretical grounds only are absurd. I have seen the infected youths at that hospital (pointing in the direction of the hospital) and the cause of their misery is not disputed. How ridiculous to deny in London what every one here knows to be true." He described his efforts to obtain access to the official reports in Paris, but Dr. Dujardin-Beaumetz, the surgeon-in-chief at that hospital, declined all information, but suggested that he obtain an authorization from the general in command, and one of the physicians said: "The best way to do this will be by means of an introduction from the British Consul."

Surgeon-Major Brassy, the Military Secretary, said: "This affair has been bad for the army, bad for vaccination, and bad for us, and I do not think you will obtain the authorization required."

Mr. Tebb called at the British Consulate and stated to Colonel Playfair (brother to Dr. Sir Lyon Playfair) what had occurred at the Hòpital du Dey, and Colonel Playfair positively refused to give the letter or to aid him in any way to get the information required! Mr. Tebb went to Algiers, and on Sunday, March 23d, 1884, had an interview with the general in command pursuant to an appointment solicited by him.

Qs. 9,533-4. Mr. Tebb begins a narrative of what took place, refreshing his memory from his notes taken at the time.

Q. 9,549. The colonel (Colonel Gaussard) stated he was authorized to give him the facts in brief, and then read from a paper prepared not by but for him: "The recruits of the Fourth Regiment of Zouaves were vaccinated according to military regulations on December 30th, 1880, at the Hôpital du Dey; fifty-eight of them were operated upon with lymph from a Spanish child of *remarkably healthy* appearance, each soldier being vaccinated in six punctures. The whole were infected with syphilis, the surgeon having mistaken syphilitic pustules for vaccine vesicles."*

On Wednesday, June 11th, 1890, Mr. Tebb's examination was resumed.

Q. 9,733. Gives from a journal, *L'Akhbar*, of August 1st, 1881, the following description by a physician: "At the expiration of one month a superficial cicatrization obtained by

---

* This statement prepared for the Colonel by medical officials who, by their position, were pledged to uphold vaccination, and ingenuously stated by the English vaccinating official, Dr. May, were anxious, as he was, "to save vaccination from reproach," deserves some notice. To save vaccination, the doctor who vaccinated is to be sacrificed. He is said to have mistaken "syphilitic pustules for vaccine vesicles;" but as shown in the pathologiccal table published in THE HOMŒOPATHIC PHYSICIAN of June, 1898 (vol. xviii, pp. 239-45), the vaccine vesicle is a chancre. The Spanish child was of remarkably healthy appearance until some time after it was vaccinated, and there can be little doubt in the mind of any person of plain common sense, whether physician or layman, but that syphilis was invaccinated upon it. It died of syphilis, as did several of the soldiers.—M. R. L.

the first treatment allowed the greater number of those who had entered to leave the hospital. Only six remained, but the others were not long in returning, in consequence of the serious symptoms which manifested themselves in every case in the most dangerous manner, with differences due to various temperaments.

"In a comparatively short period an absolute constitutional syphilization had invaded all the organs. Every patient showed more or less all the symptoms of syphilis. We have observed and recognized the following: Ulcerated wounds on the arm, cicatrized according to the individual at the end of three, four, or five months. There is one among them with whom they are not yet healed. Roseola, mucous patches on the lips, the tongue, the palate, the pharynx, etc. Indurated chancres, with or without gonorrhœa, swelling, boils and blotches on all parts of the body, copper spots, sore throat, receding gums, toothache, violent headache, enlargement and inflammation of all the ganglions of the lymphatic system, in the jaws, the neck, the throat, the joints of the loins, the arms and the legs, falling off of the hair, the eyebrows, and the eyelashes."

Q. 9,734. The Chairman asks: "Would you say for what purpose you are reading this?"

Q. 9,736. The editor of *Le Petit Colon*, the first journal which published an account of the disaster—Mr. Charles Marchal, who is also Secretary to the Conseil-General of the city, stated to Mr. Tebb: "To describe their condition is impossible; in one word, they are ruined for life." He also informed Mr. Tebb that he knew that after terrible sufferings one-half the soldiers had succumbed to the terrible disease. He then described the satirical comments of the Algerian papers on the statement of Mr. Dodson in the British House of Commons pretending that he could not get any information from the French Minister of War, and accusing the French military doctors of having neglected to inquire into the health of the vaccinifer. Also refer to the disaster at Leburs, in Wirtemburg, where on July 1st, 1876, twenty-six young girls were vaccinated from an infant of seven months, apparently in excellent health. From four to six weeks after the operation twelve of the young ladies exhibited indisputable symptoms of syphilitic disease.

Qs. 9,740-46. Professor Michael Foster and Mr. Meadows White take hold of the statement prepared for Colonel Gaussard, that the surgeon had "mistaken syphilitic pustules for vaccine vesicles," and try to make out that the fault lay with the surgeon. They fail entirely in that attempt.

Q. 9,747 (By Dr. Collins). It was in consequence of the incompleteness of the information he was able to obtain from official sources here that he went to Algiers personally.

Q. 9,748. Mr. Dodson here said that so far from admitting the fact that this disease was communicated to the vaccinifer, he could not entertain the slightest doubt that such was not the case, the more especially as the children from whom the lymph was taken were in excellent health.

Q. 9,749. The editor of the *Journal d'Hygiene*, Dr. de Pietra Santa; General Farre, the Minister of War, and Colonel Gaussard all said that the children appeared in excellent health.

Q. 9,751 (To Dr. Collins). Sir John Simon, in answer to Q. 3,523 before the Select Committee of 1871, said: "There would be no danger in taking vaccine lymph from a child having an hereditary taint as yet undeveloped."

Q. 9,752. There was no rash upon the child.

Q. 9,754. There was no evidence whatever from any source tending to show that the vesicle which furnished the lymph was a syphilitic pustule, except the fact that the fifty-eight men who were vaccinated from it had syphilis. (See note to Q. 9,549, supra, p. 389.)

Q. 9,755-62. Questions by the Chairman, Sir Wm. Savoy, and Professor Michael Foster who try to confound Mr. Tebb on this point, but with the effect of making it come out all the stronger from various confirmations.

Q. 9,763. Sir James Paget asks Mr. Tebb, who is not a physician, "Is there any form of syphilitic pustule which is at all like a vaccine vesicle?" But Mr. Tebb says he does not know. (As shown in the table before referred to the vaccine vesicle is a chancre, but generally a very mild one; occasionally it assumes its normal virulence.)

Q. 9,766 (To Dr. Collins). Dr. Dujardin-Beaumetz told Mr. Tebb at the Hôpital du Dey that he had all the facts of the disaster there. "So that," said Mr. Tebb, "they sent me to the wrong man." (Was that done intentionally?)

Qs. 9,767-84. Further quibbling over Colonel Gaussard's statement.

Q. 9,785. Mr. Tebb, as an answer to a question of Dr. Collins before put, quotes Dr. Ballard's prize essay, p. 344: "But is the syphilitic contamination of the puncture always pustulous? Does it never happen that the vaccine virus and the syphilitic virus are each drawn at the same moment from the same vesicle, and that, too, a fine, perfect, complete, and unmistakable Jennerian vesicle? I believe that it does happen. I believe it happened in the Rivalta and the Lupara series; and this disposes again of the theoretical objection that the same vesicle cannot furnish both viruses, unless it be modified somehow in its characters. The perfect character of the vesicle is no guarantee that it will not furnish both vaccine and syphilitic virus." Dr. Ballard, says Mr. Tebb, is one of Her Majestiy's Inspectors of Vaccine.*

Qs. 9,786-96. Questions directed to ascertain the character of the evidence showing the vaccinfer to have been healthy, at least in appearance, up to the time of its being vaccinated. The result is to show there is no reasonable doubt but that the child was perfectly healthy up to that time.

*The truth of the matter was so plain that these learned men could not see it. Jenner's Vaccinia and Syphilis are one.—M. R. L.

Q. 9,797. As to the disasters at Rügen. A serious outbreak of a peculiar skin disease. In consequence of serious and fatal effects following vaccination in Germany the Government has established central vaccine institutes to introduce a safer variety of vaccine. As the result of his inquiries Mr. Tebb received an official communication from Dr. Med. Koehler, Regierungs and Medical Rath, in which that gentleman states, under date Stralsund, October 29th, 1885:

"On the peninsula Wittow, Isle of Rügen, seventy-nine children were vaccinated on June 11th with humanized thymos lymph obtained from the vaccine institute, and with three exceptions all fell ill in the second week after the vaccine operation of a pustular eruption (ausschlag)." The three who escaped seem from the report to have been twelve years old or over.* "By the vaccinated children, brothers, sisters, and parents became infected, and the number of the sufferers rose to 320 out of the 5,000 inhabitants. About three-fourths have recovered, the remainder are still ill." (The document making this report is dated Stralsund, October 29th, 1885). "The disease was conveyed by the vaccinations, but the children from whom the lympth was taken were (according to the most careful and searching official inquiries) found free from eruptions, and the original cause of the outbreak is not ascertained. The complaint has been named by the English Dr. Foss (Dr. Fox), 'Impetigo contagiosa.' This is a simple infectious but not dangerous disorder. No adult has died of it."

On the attention of the Local Government Board being called to the case, after an inexcusable delay from May 20th to June 28th, after various urgings from members of Parliament, the Local Government Board caused its Assistant Secretary, S. B. Provis, to write to Mr. A. D. Connor, M. P., a

*The *influence* of *age* corroborating the opinion expressed by me in the note to Dr. Cory's testimony (HOMŒOPATHIC PHYSICIAN, Vol. xviii, June No., p. 239).—M. R. L.

letter, in which occurs the following: "I am to add that the evidence connecting the disease with the operation, which professed to be vaccination, is, in the opinion of the Board's medical officer, inconclusive, and also that the operation was not 'vaccination' as the word is understood in England, but consisted of insertion into the arm, after the manner of vaccination, of a mixture of vaccine-lymph, thymose-solution, and glycerine, of which mixture by far the largest part must have been glycerine."*

The official German report, published in the journal of the Imperial Sanitary Commission, Nos. 24 and 26, December 15th and December 30th, 1885, is not in agreement with the official letter of the Assistant Secretary of the British Local Government Board. The German Commission's report gives a minute history of the case, and says: "The Commission are unanimously of the opinion that the outbreak of the disease has been a direct consequence of vaccination. They agree with perfect certainty that by vaccination infectious matter has been transmitted, by which the disease was in the first place caused among a number of the children vaccinated for the first time." From Q. 9,798 to Q. 9,834 the Chairman, Dr. Bristowe, Professor Michael Foster, Sir James Paget, and W. Hutchinson labor hard with the witness to show that the German vaccination was not real vaccination, and that it was the glycerine which caused the mischief. This is explicitly stated by Lord Herchel at Q. 9,798 as being the contention of the Local Government Board. This intention becomes amusing, if anything in the awful tragedy of the vaccination superstition can be amusing.

---

*This is the mixture which the English Local Government Board is now seeking to force upon the people of England in the place of the "arm-to-arm lymph" which they heretofore compelled public vaccinators to employ, on pain of being refused the "extra allowance" for good work. —M. R. L.

